# Transcatheter Valve-in-Valve Aortic Valve Replacement in Stented Coarctation With Restenosis: A Case Report

**DOI:** 10.1016/j.jscai.2025.103921

**Published:** 2025-09-09

**Authors:** Pramod Sagar, Aashish Chopra, Kothandam Sivakumar, Mullasari Sankardas Ajit

**Affiliations:** a Department of Pediatric Cardiology, Institute of Cardiovascular Diseases, Madras Medical Mission, Chennai, Tamil Nadu, India; b Department of Cardiology, Institute of Cardiovascular Diseases, Madras Medical Mission, Chennai, Tamil Nadu, India

**Keywords:** bicuspid aortic valve, case report, coarctation of aorta, coarctation stent angioplasty, transcatheter aortic valve replacement

## Abstract

Combination of bicuspid aortic valve and coarctation of aorta (CoA) is common. With the widespread use of transcatheter aortic valve replacement (TAVR), cases associated with either untreated or treated CoA forms an important subset and pose specific challenges. We discuss a case of a patient with previously stented coarctation presenting with degenerated aortic bioprosthesis considered for TAVR due to porcelain aorta. The CoA stent was significantly underexpanded, which was balloon dilated, followed by TAVR with a self-expandable valve. We discuss the factors determining sequence of management, choice of CoA management, access and valve choice for TAVR in this subset.

## Introduction

Bicuspid aortic valve (BAV) is present in 60% to 80% of patients with coarctation of the aorta (CoA), with variable severity of aortic stenosis or regurgitation.^1^^,^^2^ Patients may present from infancy to late adulthood, and progressive valve disease can occur after prior CoA interventions.^3^, ^4^, ^5^ Proximal aortic valve stenosis can mask the severity of CoA by reducing flow, leading to underestimation of its hemodynamic significance. Relief of valve stenosis during surgical or transcatheter aortic valve replacement (TAVR) may unmask a hidden coarctation gradient, complicating recovery.^4^ This report discusses a case of CoA with significant luminal stenosis after a previous stent angioplasty, with a degenerated aortic bioprosthesis requiring TAVR.

## Case report

A 58-year-old woman with a history of BAV with severe aortic regurgitation and CoA diagnosed at 42 years of age underwent aortic valve replacement with a 25.0-mm Carpentier-Edwards bioprosthetic valve (Edwards Lifesciences). Surgical CoA repair was not performed due to extensive collaterals, and CoA stent angioplasty was performed using 8-zig 34.0-mm covered Cheatham-Platinum stent (NuMED) expanded up to 12.0 mm with complete resolution of the gradient.

Sixteen years later, she presented with progressive dyspnea and heart failure. Blood pressure discrepancy between upper and lower limbs (30 mm Hg) suggested recoarctation. Echocardiography showed bioprosthetic valve degeneration with severe low-flow, low-gradient aortic stenosis (peak gradient, 60 mm Hg; mean, 32 mm Hg), moderate aortic regurgitation, dilated left ventricle, and reduced left ventricular ejection fraction (38%). The predicted perioperative mortality by Society of Thoracic Surgeons (STS) risk calculator and EuroSCORE II was 2.99% and 5.96%, respectively, and the calculated risk of morbidity and mortality was 13.4% by STS risk calculator.

**Figure 1:**
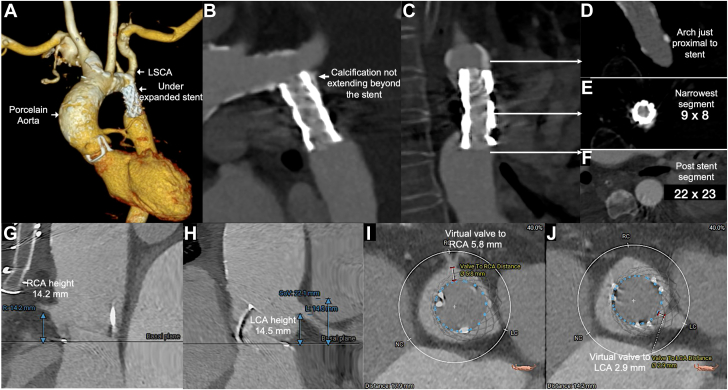
**Cardiactomography aortogram.** (**A**) A 3-dimensional reconstructed model of aorta showing diffusely calcified porcelain ascending aorta (arrow) with extension to arch. The coarctation stent is not fully expanded (arrow) extending from the isthmus to nonstenotic segment of proximal descending thoracic aorta. (**B**) Speckle of calcification (arrow) is noted posterior to the stent just beyond the left subclavian artery (LSCA) and is fully covered by the stent. (**C, D**) The calcification cranial to stent is localized to lateral walls of the distal transverse arch and are unlikely to interact with balloon dilation. The narrowest internal diameter of the stent was 8.0 × 9.0 mm (**E**) and the aorta distal to stent was 22.0 × 23.0 mm (**F**). Computed tomography assessment of aortic valve: computed tomography scan showing right coronary artery (RCA) height of 14.2 mm (**G**), left coronary artery (LCA) height of 14.5 mm, sinus of valsalva (SOV) height of 22.1 mm (**H**), virtual valve to right coronary artery of 5.8 mm (**I**), and virtual valve to left coronary artery of 2.9 mm (**J**). L, left coronary artery, LC, left coronary cusp, NC, non coronary cusp, R, right coronary artery, RC, right coronary cusp.

Although repeat surgery was planned, surgery was aborted due to dense mediastinal adhesions and heavily calcified ascending aorta (porcelain aorta), precluding safe cannulation after sternotomy. A contrast-enhanced computed tomography scan confirmed extensive aortic calcification, a degenerated bioprosthesis with a true internal diameter of 21.7 mm. The coronary heights for left coronary artery was 14.5 mm and right coronary artery was 14.2 mm, and virtual valve-to-coronary distances were 2.9 mm and 5.8 mm for the left and right coronary arteries, respectively. The maximum ascending aortic dimension was 38.0 mm, and there was no coronary artery stenosis (Figure 1). Both femoral arteries measured around 5.0 to 5.5 mm, allowing transfemoral access. As a self-expanding transcatheter heart valve (THV) would easily navigate through a CoA stent without metal to metal interaction, it was preferred. Supra-annular positioning in our patient with small annulus also favored a self-expanding THV. The only balloon-expandable THV available in our institute was Myval (Meril Life Sciences), but it was not suited for the femoral arterial dimensions.

The stented CoA segment had a 9.0-mm lumen and was calcified posteriorly near the left subclavian artery origin. The transverse arch measured 19.0 mm, and the descending aorta was 16.0 mm at the diaphragm level. Redilation of the previously implanted covered stent was considered feasible as well as the right choice for relief of the coarctation.

We considered single-stage procedure as the patient had an aborted surgery and was on mechanical ventilation and inotropic support. The alternative access options of carotid and axillary access were assessed and found to have bilateral carotid artery stenosis. The coarctation stent seemed easily amenable for balloon dilation, followed by TAVR.

**Figure 2:**
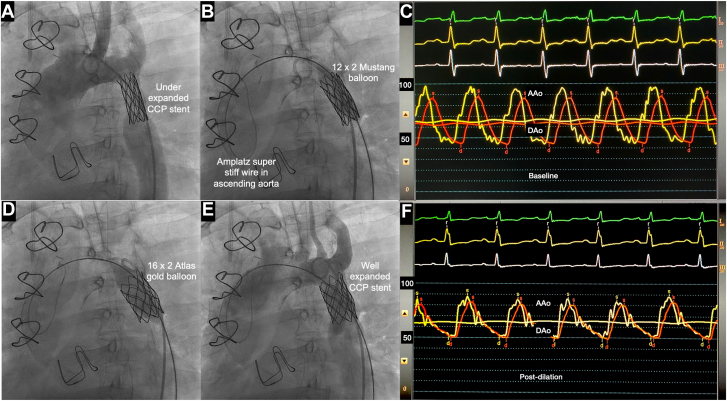
**Procedural images of coarctation stent redilation**. (**A**) Initial angiogram showing incompletely expanded stent and the origin of left subclavian artery. (**B**) Balloon dilation of stent using 12.0- × 20.0-mm Mustang balloon. (**D**) Final expansion with a 16.0- × 20.0-mm Atlas gold balloon. (**E**) Angiogram confirming no dissection or rupture. (**C**, **F**). Simultaneous ascending aortic (AAo) and descending aortic (DAo) pressure trace showing a peak-to-peak gradient of 10 mm Hg and nearly equal pressures after balloon dilation (**C, F**). CCP, covered Cheatham-platinum.

Under general anesthesia, right femoral arterial access was used for both stent redilation and TAVR, with left femoral arterial access for angiography and venous access for rapid pacing. CoA stent dilation was performed sequentially using 12.0 × 20.0-mm Mustang (Boston Scientific) and 14.0 × 20.0-mm and 16.0- × 20.0-mm Atlas gold balloons (Bard Peripheral Vascular). The CoA gradient of 10 mm Hg was abolished, and angiography confirmed no aortic injury (Figure 2).

**Figure 3:**
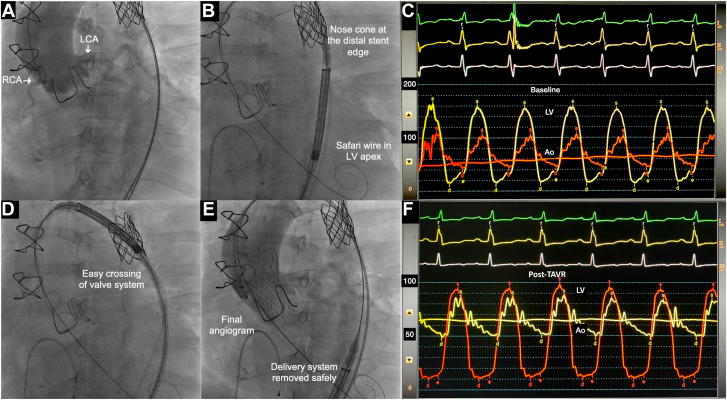
**Proceduralimages of transcatheter aortic valve replacement (TAVR).** (**A**) Aortic root angiogram showing the coronary artery origins (**A**). Evolute valve proximal to the coarctation stent (**B**), advancing the valve assembly across the coarctation stent (**D**) and final valve position (**E**). Preprocedural simultaneous left ventricle (LV) and ascending aortic pressure (AAo) traces showing a peak-to-peak gradient of 60 mm Hg, reduced to 10 mm Hg after TAVR (**C, F**).

The bioprosthetic valve was crossed using a 6F Amplatz Left 1 catheter (Medtronic) and a 0.035-inch glide wire, which was exchanged for a Safari 2 extra-small curve wire (Boston Scientific). Hemodynamic evaluation showed a 60 mm Hg peak systolic gradient across the valve. A 26.0-mm Evolut-R valve (Medtronic) was advanced across the redilated CoA stent without difficulty and deployed within the degenerated bioprosthesis under rapid ventricular pacing. Fluoroscopy confirmed stable valve position; aortography showed no aortic regurgitation and patent coronary arteries. The post-TAVR peak-to-peak gradient reduced to 10 mm Hg. Vascular closure was achieved with a single device (Figure 3).

**Figure 4:**
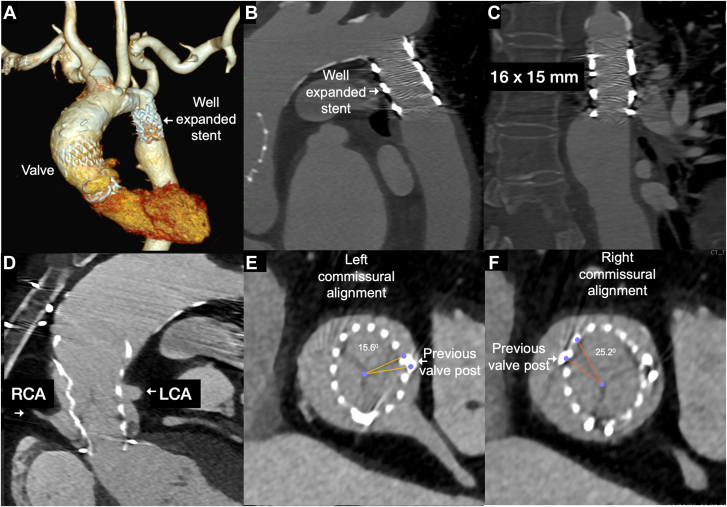
**Postprocedural computed tomography.** (**A-C**) Fully expanded coarctation stent (arrow), no pseudoaneurysm and well flowing left coronary artery (LCA, arrow) and right coronary artery (RCA, arrow) through the valve frame. The commissural alignment to the previous valve posts (arrow) shows normal alignment of left (**E**) and mild misalignment to right (**F**).

The patient was extubated on postoperative day 2 and discharged on day 8 with dual antiplatelet therapy. At 1-year follow-up, she remained asymptomatic. Blood pressure was equal in all limbs, echocardiography showed a mean transvalvular gradient of 6 mm Hg, trivial aortic regurgitation, and improved left ventricular ejection fraction of 59%. Computed tomography confirmed good apposition of the CoA stent without pseudoaneurysm and unobstructed coronary arteries. Commissural alignment was assessed and right was found to be mild misalignment and left found to be normal alignment to the previous bioprosthetic valve posts (Figure 4). In case of a need of future TAV in TAV if the valve degenerates, we may have to consider leaflet modification with available techniques during the time of procedure.

## Discussion

Simultaneous presentation of aortic valve disease in BAV with significant CoA requires a coordinated approach. In adults, covered stent angioplasty is the preferred strategy for treating CoA. The choice between surgery and TAVR depends on perioperative risk, with TAVR favored in intermediate-risk to high-risk patients.^6^ In patients with prior CoA stenting, transfemoral TAVR may result in device–stent interactions. If both interventions are required, TAVR should precede CoA stenting to reduce such risks. However, in previously stented patients, full reexpansion of the CoA stent should be performed before TAVR to ensure smooth passage of the delivery system.

Three clinical scenarios are seen with CoA and aortic stenosis: (1) untreated severe CoA with valve disease, (2) repaired or stented CoA without residual disease, and (3) repaired/stented CoA with residual stenosis. In untreated CoA, if the TAVR system can cross the coarctation, stenting may follow valve deployment. For critical CoA with near-total lumen obliteration, staged procedures, initial stenting followed by later TAVR, may be safer as reported by Marchese et al^4^ and Fallatah et al,^7^ where, in both cases, the CoA stenting and TAVR were separated by 1 week. The case reported by Marchese et al^4^ was more challenging for TAVR as the aorta was horizontal and CoA stenting was performed only 1 week prior to TAVR, positing challenges in delivery of self-expanding valve and was achieved using snare assistance.^4^ In this case, the stent was deployed almost 16 years prior and the risk of stent distortion was remote, and in view of residual stenosis limited to the stent, segment redilation was alone sufficient, and there was no difficulty in advancing the self-expanding valve. Transfemoral CoA stenting with transcarotid TAVR in a single sitting is an alternative approach as reported by Zhong et al^8^ and He et al^9^ without interfering with the CoA stent. In this case, use of snare assistance or use of alternative access including carotid or axillary access could have been the bailout strategies as described in earlier reports.^4^^,^^8^^,^^9^ In previously treated CoA, imaging should assess for aneurysms, stenosis, or calcification. Recoarctation can often be managed with stent reexpansion. However, disease beyond the original stent may require longer covered stents. In this case, low gradients across a narrowed stented segment (9.0-mm lumen) were likely due to anesthesia, severe proximal valve stenosis, and left ventricular dysfunction.

Self-expanding TAVR valves with fully covered sleeves are preferred in the setting of CoA to minimize device–stent interaction. However, balloon-expandable valve with deflectable delivery catheter can also cross the stent frame easily. Poststenotic dilatation in the descending thoracic aorta requires careful navigation of the TAVR delivery system through the stented segment to prevent complications.

## Conclusion

Combination of significant aortic valve disease and CoA needs a planned strategy in adults where transcatheter modes of treatment may be feasible. Treatment plan depends on age of presentation and severity of individual lesions. Balloon dilation of stented CoA with restenosis is important to avoid interactions between the stented segment and TAVR delivery system.
